# Supplementary material for: Learning curve and surgical outcome of robotic assisted colorectal surgery with ERAS program
Source: Sci Rep. 2022 Nov 29;12:20566. doi: 10.1038/s41598-022-24665-w (PMC9709162; doi:10.1038/s41598-022-24665-w)
Supplement: Supplementary file 2 — Supplementary Information 2. [file 41598_2022_24665_MOESM2_ESM.zip › RAL Raw data-Ñ[▒K/059/HS_ñw╝╨░O▒Kñσ.pdf]

正式摘要(電子簽章：0000020974O896)

W52 / W52 ☐ 英文標題(注意:無英文姓名-請補病人基本檔!) ☐ 【讀取】憑證

臺中榮民總醫院

出院病歷摘要

轉入號 : 轉出號 :

[redacted]

**(8)**流水編號	
**(9)**入院日期	110/01/29 (2021) 直腸外科 W52 - 112
**(10)**轉科日期	
**(11)**出院日期	110/02/03 (2021) 住院天數計 5 日

診斷

- 入院 :adenocarcinoma of sigmiod colon, cT3N1M0
- 出院 :adenocarcinoma of sigmiod colon, cT3N1M0 s/p robotic AR on 1/30

主訴

- bloody stool for about 4 months

病史

- This 52 years old male patient didn't have special past history. Bloody stool was noted since 4 months ago. Thinner stool, lower abdominal pain and sometimes urinary urgency were also noted. He came to LMD for help. Sigmoid colon tumor 20cm from anal verge was found and malignancy was proved. He came to our hospital for help. In abdominal CT, sigmoid colon tumor and parapancrease tumor were found. MRCP was done during last admission and showed a big septated cystic lesion at right anterior pararenal space, about 77x60x48mm, locating behind the 2nd and 3rd portion of duodenum and pancreatic head. Lymphangioma was ruled in. This time, he was admitted for surgical treatment.

體檢發現

-
    - General appearance :
        - easy looking , conscious clear , without cardiopulmonary distress
    - HEENT :
        - Eyes: no anemic conjunctiva, no icteric sclera, normal EOM
        - ENT: no otorrhea, no rhinorrhea, no erythematous mucosa, no injected tonsil, no deformity
    - Neck: supple, freely movable, trachea midline,no deformity, no engorged jugular vein , no palpable thyroid or lymph node enlargement
    - Chest:
        - Inspection: Grossly normal, symmetric and fully expansion, no accessory muscle used, no operation scar, no spider angiomata, no gynecomastia
        - Palpation: no palpable mass or nodule, no subcutaneous emphysema, no tenderness
        - Ausculation: clear breathing sound
    - Heart:
        - Percussion: normal heart size
        - Ausculation: regular heart beat, normal S1, S2, no gallop, no murmur
    - Abdomen:
        - Inspection: in
            - flat contour, symetrically, no scar, no stria, no dilated veins, no rashes
        - Ausculation: normoactive bowel sound, no abdominal bruits
        - palpation: soft, no muscle guarding or rigidity, no local tenderness, no rebounding pain, no palpable mass, no palpable pulsation
        - Percussion: no shifting dulllness, no hepatomegaly, no splenomagaly
    -

Peripheral pulsation :	CA	BA	RA	FA	PA	PTA	DPA
Right side :	++	++	++	++	++	++	++/+++
Left side :	++	++	++	++	++	++	++/+++

  - Genito-anus: no tenderness or palpable mass lesion or rectal shelf, normal external genitalia, normal anal tone, brown stool

CRS CONDITION

-
  - Lung

手術日期及方法

- 手術日期及方法20210130 1. Robotic (XI) assisted anterior resection
  2. Intraoperative colonoscopy : (-).
  3. High ligation (-) : IMA, left colic artery and IMV were also ligated via Hemoloks.
  4. Take down from the splenic flexure : (-)
  5. Robotic endoGIA (45-4.3) X1 + EEA (33mm) were used
  6. Proximal cut end: 2 cm, distal cut end: 5 cm
  7. No drainage tube was inserted

住院治療經過

- After admission, operation was done on 1/30, after the operation, the patient try liquid diet and tolerate well. He had stool passage. Under stable condition, the patient was discharge with OPD follow up.

合併症

一般檢查

特殊檢查

病理報告

其它

出院時情況

- 3.改門診治療

出院指示

藥名	劑量	劑量單位	用法	頻次	數量	數量單位	現況	天數	單位
Ultracet tab	1	TAB	PO	Q6H	28	TAB	TH	7	天
(□)Takepron OD tab 30mg	1	TAB	PO	QDAC	7	TAB	TH	7	天

出院計劃

主治醫師 2062F 蔣鋒帆 / 住院醫師 2332G 林志安

正式摘要(**電子簽章：000000209740896)**

W52 / W52

doctorRank:B
